# Words and Worlds Both: Dynamic Effects of Distributional and Sensorimotor Information in Semantic Processing

**DOI:** 10.1162/OPMI.a.316

**Published:** 2025-12-18

**Authors:** Harshada Vinaya, Sean Trott, Diane Pecher, René Zeelenberg, Seana Coulson

**Affiliations:** Department of Cognitive Science, University of California, San Diego, San Diego, CA, USA; Department of Psychology, Education, and Child Studies, Erasmus University, Rotterdam, Netherlands; Kavli Institute for Brain and Mind, San Diego, CA, USA

**Keywords:** semantic memory, distributional semantics, embodied semantics, Lancaster sensorimotor norms, EEG

## Abstract

An important issue in the semantic memory literature concerns the relative importance of experience-based sensorimotor versus language corpus-based distributional information in conceptual representations. To explore how each contributes to behavioral and neural responses on a conceptual task, EEG and RTs were recorded as healthy young adults viewed terms for concepts (e.g., “APPLE”) followed by properties (e.g., “red”) and pressed a button to indicate whether the property is true or false for the concept. Next, we constructed a series of mixed effects models of response times (RTs) and single-trial electroencephalogram (EEG) responses to the property words. Distributional models predicted data using semantic distance measures (e.g., between “APPLE” and “red”) derived from language corpus-based measures developed by computational linguists. Sensorimotor models predicted data using sensorimotor distance, a measure based on comparisons of each word’s experiential strength on the perceptual and action-effector dimensions from the crowd-sourced Lancaster Sensorimotor Norms. Statistical model comparison was used to determine whether the data was best fit by Distributional, Sensorimotor, or both sorts of information. In keeping with hybrid accounts of semantic memory, we find that both measures of semantic distance explained unique variance for behavioral and neural measures. Modelling EEG across seven successive 100-ms intervals revealed that the predictors’ temporal dynamics varies between true (APPLE – red) and false (APPLE – black) trials, but showed early sensorimotor activation for both. Results show how linguistic context and task demands modulate the recruitment of different information sources, supporting dynamic hybrid accounts of semantic memory.

## INTRODUCTION

The study of semantic memory pursues fundamental questions about human cognition, such as how we recognize, classify, and utilize our knowledge of objects and events, as well as how we comprehend and convey meaning through language. Theories of human semantic memory have often contrasted the assumptions and predictions of distributional approaches to meaning with those of embodied or grounded accounts (Davis & Yee, 2021). Distributional theories propose that the meanings of words can be derived from their linguistic distributions, i.e., the words with which they tend to co-occur (Harris, 1954). For example, the words “apple” and “sweet” might become related as they frequently occur with each other, while the words “apple” and “peach” might become related as they each occur with words like “sweet”, “juicy”, and “round”. Distributional theories suggest meaning is exclusively derived from statistical patterns of word co-occurrence, and decades of research supports their cognitive feasibility by showing that measures derived from language co-occurrence data align with human behavioral performance on semantic tasks (Harris, 1954; Lenci, 2018; Mandera et al., 2017). Embodied or grounded frameworks, by contrast, underscore the significance of sensorimotor or experiential information in our conception of meaning. Note that theories of grounded semantics vary widely, with some incorporating not just the sensorimotor system, but also our affective experiences and the environment, broadly suggesting that language comprehension recruits information deriving from experiences and events related to the corresponding words (Barsalou, 2015; Barsalou et al., 2003; Hauk & Tschentscher, 2013; Meteyard et al., 2012).

In recent years, a number of “hybrid” proposals have attempted to reconcile these approaches, arguing that both sources of information contribute to our semantic knowledge. Among the hybrid accounts, there is general agreement that grounded and distributional information each play a role in semantic representations, although claims differ regarding their relevance and context-dependency (Davis & Yee, 2021; Kemmerer, 2019; Winkielman et al., 2023). For example, one of the earliest accounts, the Language and Situated Simulation (LASS) theory (Barsalou et al., 2008) suggests that the initial stage of word processing (e.g., for “apple”^1^) involves the activation of word-associates (e.g., pie, red, crispy) that act as pointers or cues to more detailed information about the underlying concept (viz., APPLE). Presumably the province of distributional semantics, these word associations’ contribution to semantic processing is proposed to peak earlier than the sensorimotor simulations that follow. The LASS account further suggests that the earlier accessed word associations alone might be sufficient to produce correct responses on many conceptual tasks (Barsalou et al., 2008). Similar observations about successful task outcomes are also made by Louwerse (2011) in his articulation of the Symbol Interdependency Hypothesis. This hypothesis goes beyond LASS, proposing that word associations not only serve as pointers, but also encode part of the sensory and motor-based world knowledge via their statistical properties, such as the fact that cats have fur increases the likelihood of “cat” co-occurring with “fur”, and likewise the fact that “lemon” tends to co-occur with “sour,” “yellow,” and “citrus” arises in part because lemons are a sour, yellow, citrus fruit (Louwerse, 2011).

The Linguistic Shortcut Hypothesis (LSH) also goes beyond LASS in suggesting that linguistic information can furnish perceptual associations for concepts that have not been directly experienced (Connell, 2019). For example, even if we haven’t flown unaided, FLYING can be simulated through its perceptual associations with motion and spatial concepts. Moreover, via word associations, linguistic information can even capture conceptual information that is not accounted for by perceptual simulation (Connell, 2019). To illustrate this, Connell and colleagues suggest that the lexical representations of democracy, freedom, and human rights result from distributional information learned through exposure to books or articles, and are content-wise distinct from the concrete experiences of participating in elections and voting (Connell, 2019; Wingfield & Connell, 2023).

These suggestions underscore the potential for unique contributions from language-distributional and grounded resources to conceptual knowledge. Wang et al. (2020) add evidence of the uniqueness of distributional and sensorimotor knowledge showing encoding of color knowledge agnostic to the availability of perceptual information. They investigated the brain regions involved in representing object-color knowledge in congenitally (and early) blind individuals compared to sighted ones. This comparison revealed that some regions support color knowledge only in the sighted, while others, such as the left dorsal anterior temporal lobe, support object-color knowledge in both blind and sighted individuals. These findings suggest color knowledge draws on brain regions that support the perception of color as well an encoding that is likely language-derived and sensory-independent (Wang et al., 2020).

Hybrid accounts also broadly suggest that contextual factors, like comprehension goals, shape the relative importance of either kind of information retrieval (Kemmerer, 2019; Kuhnke et al., 2020; Winkielman et al., 2023). Louwerse and Jeuniaux (2010) analyzed response times (RTs) from participants making speeded judgments about semantic relatedness and spatial iconicity regarding pairs of concepts, presented using either symbolic representations (words) or analogical ones (pictures). They operationalized distributional information as the frequency of word order in language (e.g., “attic–basement”, see Louwerse, 2008 for details), and sensorimotor information as the extent to which the words/pictures were arranged in a real-world spatial configuration (e.g., “attic” above “basement”), and found that sensorimotor information better predicted participants’ performance on the pictures in the spatial iconicity task, while distributional information better predicted performance on the words in the semantic relatedness task. These task-based dynamics demonstrate how context can shape the recruitment and salience of different kinds of information.

Simmons et al. (2008) investigated the neural basis of this differential information recruitment as well as the prioritization of linguistic information predicted by the LASS theory (Barsalou et al., 2008). Using functional Magnetic Resonance Imaging (fMRI), they conducted a conjunction analysis to identify overlapping brain regions activated during two different tasks performed a week apart (Simmons et al., 2008). In the first stage, participants were scanned as they performed a property generation task, which served as a measure of conceptual processing. Each trial in the property generation task lasted 15 seconds and was divided into two parts to represent the early and late phases of conceptual processing. A week later, participants returned for the second stage, where they performed two localizer tasks: one measuring word association and the other situation simulation. Interestingly, the early phase of property generation (i.e., the first 7.5 seconds of each trial) showed no overlap with situation simulation activations, but the late phase did, notably in regions linked to biological motion perception (right posterior superior temporal sulcus), episodic memory (posterior cingulate), and mental imagery (bilateral precuneus). These findings support the LASS theory, suggesting that later conceptual processing engages brain regions involved in various aspects of sensory and experiential engagement with the world (Simmons et al., 2008). However, due to the temporal resolution of the BOLD response, their data provide only limited support for claims regarding the timing of the neurocognitive events in conceptual processing. A more robust test of the temporal prioritization proposal in LASS would require a measure with millisecond-level sensitivity.

Accordingly, in the present study we utilize response variables with greater temporal resolution by measuring electrical brain activity via scalp-recorded electroencephalography (EEG) and response times (RTs) collected as participants performed a conceptual task. We compare the timing of the engagement of grounded versus language distribution based neural systems by examining EEG and RTs collected during the property verification task. In this task, participants are successively presented with word pairs composed of concepts (“APPLE”) and properties (“red”) and asked to make decisions about whether the properties are typically mapped to the concepts. The property verification task has been widely used in the grounded semantics literature due to its presumed engagement of sensorimotor processes to simulate the objects (i.e., APPLE) in order to report whether the property (RED) applies (Pecher et al., 2003). Even so, because property verification is often performed under instructions of speed, some researchers speculate that participants may also (or alternatively) recruit distributional information (Connell, 2019; Solomon & Barsalou, 2004). While verifying that RED is a property of APPLE might involve using information derived from the sensory representation of apples, it might also reflect the associative strength between the two words, e.g., how frequently the words “APPLE” and “red” co-occur.

This has been a contested issue for the property verification task, with previous work showing that participants use different strategies based on context and instructions. For instance, Solomon and Barsalou (2004) manipulated two variables between subjects during this task. First, they varied whether the FALSE trials were lexical associates (e.g., “BUFFALOES” – “winged”) with high co-occurrence in language or not (e.g., “APPLE” – “black”). This manipulation was intended to discourage participants from responding TRUE whenever the two words sounded good together rather than retrieving the relevant knowledge from semantic memory. Second, they varied the instructions, asking half of the participants to rely on mental imagery and giving the other half neutral instructions. Modeling the response times and error rates using perceptual and linguistic predictors, they found that perceptual predictors performed better both with imagery instructions and when FALSE associates (e.g., “BUFFALOES” – “winged”) were included (Solomon & Barsalou, 2004). Kan et al. (2003) examined the same proposal with fMRI, using the brain response in visual association regions as an index of the use of perceptual information. Like Solomon and Barsalou (2004), they manipulated whether the experimental trials (e.g., “BEAR” – “brown”) were presented along with associated FALSE trials (e.g., “CAT” – “litter”), intended to discourage the use of shallow associative strategies, or with unassociated ones (e.g., “CAT” – “wine”). Kan and colleagues found that the recruitment of visual cortical regions implicated in mental imagery occurred only when experimental trials were accompanied by the associated FALSE trials (e.g., “CAT” – “litter”). Overall, this suggests participants recruit both linguistic and perceptual information to perform the property verification task, making it a good testbed for evaluating the importance of distributional and sensorimotor information during conceptual processing.

Capitalizing on recent advances in natural language processing and online data collection to quantify the semantic distance between, for example, “APPLE” and “red”, here we evaluate the relative contributions of each to behavioral and neural data collected during this task. Naturally, a key challenge concerns how to operationalize these information structures, i.e., which representations to use (and how) as proxies for distributional and sensorimotor information. In the distributional semantics literature, researchers often represent semantic content using multidimensional vectors derived from large text corpora, such as books, articles, and web pages, and capture patterns of word co-occurrence (Mikolov et al., 2013). In keeping with the core principles of distributional semantics, vectors of words that frequently occur in similar contexts are mapped closer to each other in this high-dimensional meaning space. This method has allowed investigators to quantify semantic similarity, analyze the underlying structures and relationships between words, and even to test these models against human semantic performance (Mandera et al., 2017).

Following this vector-based approach, we operationalize distributional information as the semantic distance measured between concept—property pairs (e.g., “APPLE” – “red”) using the GloVe or Global Vectors for Word Representation embeddings (Pennington et al., 2014). Like other count-based models, the GloVe model starts with word co-occurrence probabilities but then uses ratios between these probabilities to form vectors. Borrowing the example from Pennington et al. (2014), if we are working with words “ice” and “steam”, first their relationships with context words like “solid,” “gas,” “water,” and “fashion” are examined through co-occurrence probabilities. After that, the model captures the ratios of co-occurrence probabilities to find that the context word “solid” is more likely to occur with “ice” than with “steam”, and conversely, “gas” occurs more with “steam” than “ice”. The model leverages the ratios of probabilities to build word vectors that better capture the semantic distinctions compared to the raw probabilities. The GloVe model has been used to evaluate distributional models for human semantic effects using both behavioral and neural data, including semantic similarity judgments (Richie & Bhatia, 2021) and the amplitude of the N400 component in the event-related brain potential (ERP) elicited by words in sentences (Michaelov et al., 2024).

Here we extend the vector based approach to operationalize the sensorimotor information for the words in our property verification task using the Lancaster Sensorimotor Norms (Lynott et al., 2020; Wingfield & Connell, 2023), one of the crowd-sourced set of human ratings capturing the perceptual and motor associations of words (see also Binder et al., 2016). In the Lynott et al. (2020) study, participants were given words such as “apple” and asked to rate the extent their knowledge of the term relied on each of six perceptual modalities (vision, audition, gustation, olfaction, touch, and interoception) or interaction with five action effectors (foot/leg, hand/arm, torso, mouth/throat, and head excluding mouth), and the dataset presents ratings from 3,500 participants who assessed 39,707 words across the mentioned 11 dimensions. Compared to feature-based or aggregate measurements such as concreteness or imageability ratings, these norms may offer a detailed reflection of the neural underpinnings of grounded semantics (Trott & Bergen, 2022).

Such measures derived from crowd-sourced experiential norms have been used to test hypotheses involving the influence of grounded information in semantic processing (Reilly et al., 2023; Wingfield & Connell, 2023). For example, Wingfield and Connell (2023) presented a vector based measure of sensorimotor distance between any two words in the Lancaster Sensorimotor Norms database. They show that their sensorimotor distance measure could model human judgments of semantic similarity better than alternative approaches such as WordNet, a distributional semantic measure, and measures based on feature models (Wingfield & Connell, 2023). Similarly, Reilly et al. (2023) operationalized semantic distances between bigrams in continuous texts in two ways: using GloVe embeddings as distributional, co-occurrence-based semantic distance (Pennington et al., 2014), and using a few dimensions from the Lancaster Sensorimotor Norms (Lynott et al., 2020) and AffectVec (Raji & de Melo, 2020) to capture the grounded semantic distance. They used these bigram semantic distances to test hypotheses about cohesion and topic flow in continuous texts.

In a similar vein, we operationalized distributional and sensorimotor information using GloVe embeddings and the Lancaster Sensorimotor Norms respectively and use multivariate regression models of single-trial EEG and RTs collected on property words during the property verification task. We infer the relative contributions of distributional and sensorimotor information to this task from the predictive strength of each measure. If participants prioritize language-based associations to judge whether or not properties hold for the concepts, we would expect the variance in our EEG and RT data to be best explained by the distributional semantic distance measured between the concept–property pairs. Alternatively, grounded approaches predict that our dependent measures would be best explained by sensorimotor information, and hybrid accounts predict contributions from both distributional and sensorimotor information.

## MATERIALS AND METHODS

### Materials

The present study utilized data collected in an unpublished companion study for Collins et al. (2011) that adapted materials from Pecher et al. (2003). The materials included 576 concept–property pairs for the property verification task. Each trial consisted of the consecutive presentation of a concept (“APPLE”) and a potential property (“red”) word. Out of the total 576 stimulus-pairs, 480 used sensory property words, including visual (e.g., “red”), tactile (e.g., “prickly”), and auditory (e.g., “loud”) words. These 480 pairs are referred to as “property pairs”. Half of those required a TRUE response (e.g., “APPLE” – ”red”), and the other half a FALSE response (e.g., “APPLE” – ”loud”). The remaining 96 pairs were lexical associates that were included to discourage participants from using word-association as a diagnostic for property verification; half of those required a FALSE response (e.g., “BUFFALOES” – “winged”), and half required a TRUE response (e.g., “ESSAY” – “written”) (Solomon & Barsalou, 2004).

Our primary goal was to investigate the explanatory power of distributional and experiential information in predicting EEG responses elicited by words. To do this, we utilized the property pairs for which word frequency scores from SUBTLEX-US (Brysbaert et al., 2012), GloVe embeddings (Pennington et al., 2014), and Lancaster norms (Lynott et al., 2020) were all available for both the concept and property words in the trial. The logarithmic word frequency of property words in the TRUE trials varied from 0.30 to 4.19 (*M* = 2.29, *SD* = 0.84), and similarly, for the FALSE trials from 0.60 to 4.06 (*M* = 2.04, *SD* = 0.76). In the upcoming section, we will describe how we operationalized the distributional and sensorimotor information from GloVe embeddings and the Lancaster Sensorimotor Norms, respectively, and report their descriptive statistics to mark their language-statistical and perceptual profiles. The cleaned EEG epochs and the code used for the following measurements are available in the OSF repository accessible at http://osf.io/6efp8.

### Distributional Distance

We used embeddings from the pre-trained 6B (Wikipedia 2014 + Gigaword 5) GloVe model with 200 dimensions to operationalize the *distributional information* (Pennington et al., 2014). As words with similar meanings tend to cluster together in vector space, we used the *cosine distance* between their GloVe vectors as a measure of *dissimilarity* for all of our trials (Landauer & Dumais, 1997; Mikolov et al., 2013; Trott & Bergen, 2021). That is, we used the singularized, lowercase variant of each concept word and property word to retrieve their vectors from the GloVe database and then measured the cosine distance between them (e.g., between the vector for “apple” and the vector for “red”).

Cosine distance is defined as 1 − $\frac{A.B}{\left\|A\|\;\|B\right\|}$ where *A* and *B* are vector representations of two words. Intuitively, a larger cosine distance corresponds to more semantically dissimilar vectors (and thus more distinct distributional patterns). For the TRUE trials, the distributional semantic distance varied from 0.45 (“OWLS” – “screech”) to 1.05 (“SUNGLASSES” – “dark”), with a mean of 0.80 and standard deviation of 0.12. For the FALSE trials, the distributional distance ranged from 0.59 (“OVENS” – “rub”) to 1.17 (“CLOTHESPINS” – “cut”), with a mean and standard deviation of 0.92 and 0.10, respectively.

### Sensorimotor Distance

We used the same principle of cosine distance measurement to capture Sensorimotor Distance using the Lancaster norms database (Lynott et al., 2020; Wingfield & Connell, 2023). To do this, we first constructed 11-dimensional semantic vectors for each of the property and concept words using all 11 of the ratings from the Lancaster norms (i.e., the six perceptual dimensions and five action ones). Then we measured the cosine distance between concept and property vectors for pairs in all trials, similar to our distributional measure. For the TRUE trials, the sensorimotor semantic distance varied from 0.00 (“SKY” – “hazy”) to 0.56 (“KEYS” – “jingle”), with mean of 0.21 and standard deviation of 0.12. And for the FALSE trials it varied from 0.01 (“HEADLIGHTS” – “opaque”) to 0.77 (“THUMBS” – “moan”), with mean of 0.29 and standard deviation of 0.16. As we used all 11 dimensions of the Lancaster norms, it is important to note that individual dimensions of our sensorimotor semantic vectors may have information overlap due to potential difficulty in parsing multimodal experience in these dimensions and the fact that two separate groups of participants provided the action and perception ratings. We, thus, measured sensorimotor distance in perception space using only the six Lancaster perceptual norms, and separately using five Lancaster action norms. Both measures were highly correlated with the sensorimotor distance based on all 11 dimensions (perception-based distance: *r* = 0.90, *p* < .001; action-based distance: *r* = 0.74, *p* < .001). Notably the action-based and perception-based measures had a weaker yet significant correlation with each other (*r* = 0.4, *p* < .001), which suggests that while the action and perception measures share some overlap, they also contain unique variance.

### Participants

Data were collected from 24 UCSD undergraduate participants. All participants were aged between 18 and 40 years old, reported normal or corrected-to-normal vision, and had no reported history of neurological or psychiatric disorders. Data from six participants contained a high number of movement artifacts in the trial blocks, as identified through visual inspection (Luck, 2014). These artifacts were found to be uncorrectable by ICA decomposition and, consequently, these participants were excluded from the analyses. The analyses here were based on the remaining 18 participants.

### Procedure

**Figure 1.**
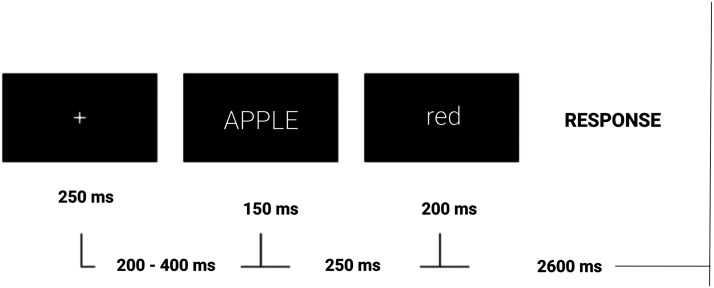
Illustration of a Property Verification Trial. Participants were presented with two words in a row. The concept (e.g., “APPLE”) appeared in capitals, followed by the property word (“red”) in lowercase. Participants’ task was to verify whether the property (e.g., “red”) was true for that concept (e.g., “APPLE”). Participants had 2600 ms to respond before being presented with the next trial.

Participants were seated in a sound-attenuating chamber about 50 inches from a 17-inch computer screen. They were instructed to read the word pairs and respond TRUE if the property was either typical or frequently possible for the concept, and FALSE if not. Participants were encouraged to respond as quickly and accurately as possible, and told that accuracy was more important than speed. Each trial began with the presentation of a fixation cross for 250 ms. Between 200 and 400 ms later, the CONCEPT term was displayed in the center of the screen for 150 ms, followed by 250 ms of blank screen. The property term was then presented for 200 ms. The 2600 ms inter-trial interval allowed plenty of time for participants to make their decision and press the button with their right hand to indicate TRUE and their left hand for FALSE. The experimental paradigm is shown in Figure 1.

### EEG Recording

EEG was recorded using a cap with 29 tin electrodes placed at standard sites from the 10–20 system manufactured by Electro-Cap International (Eaton, Ohio, USA). The montage is shown in Figure 5a. Electrodes were referenced online to the left mastoid site and later re-referenced to the average of the left and right mastoid electrodes. Blinks were monitored using an electrode below the right eye, and horizontal eye movements using a bipolar montage of electrodes placed at the right and left outer canthi. As in Collins et al. (2011), EEG was recorded and amplified using an isolated bioelectric amplifier manufactured by SA Instruments (San Diego, California, USA), with a bandpass of 0.1 to 100 Hz. The signal was digitized online at 250 Hz. All impedances were kept below 5 kOhms.

### EEG Preprocessing

The EEG data was digitally filtered using an FIR bandpass filter from 0.1 to 40 Hz. Data from all 29 scalp channels was retained as the signal quality was acceptable and channel interpolation algorithms typically require at least 64 sites (see Courellis et al., 2016 for discussion). Maximum likelihood ICA was used to identify components with eye artifacts and subtract them from the data. Components with eye artifacts were identified based on their spectral profile and topographic distribution. Because the subtraction of several different independent components can result in spurious “correction” of task-related brain activity, no more than two independent components per participant were selected for correction. EEG was timelocked to the onset of the second (property) term in each trial and epochs were created beginning 100 ms prior to word onset and ending 700 ms after. All epochs were baseline corrected using the 100 ms window before property word onset. Following artifact correction, epochs were first rejected using automatic rejection criteria of EEG voltages exceeding ±150 microvolts, and EOG exceeding ±250 microvolts, and the rest were subjected to visual inspection by a trained expert for manual rejection. The combined automatic and manual rejection rate averaged 6% across subjects. Data preprocessing was performed using implementations in the MNE package in Python (Gramfort et al., 2013). Data and code for the EEG processing is available in the OSF repository accessible at http://osf.io/6efp8.

### Analysis

Exploiting the high temporal sensitivity afforded by EEG, we modeled single trial EEG across seven successive 100 ms intervals, from the onset of the property word to 700 ms after. We chose the window of 100 ms to allow adequate temporal resolution to probe whether the relative importance of distributional versus sensorimotor distance changes in the course of processing, while avoiding a larger number of statistical tests that might be challenging to interpret (Winsler et al., 2018, 2023). We further analyzed the TRUE responses (e.g., “APPLE” – “red”) separately from the FALSE responses (e.g., “APPLE” – “black”) to circumvent the confounds introduced by differing response demands due to each sort of trial (viz., left versus right-handed responses), as well as the different cognitive demands of verifying versus rejecting a property. Separate analyses of this sort are often conducted when different responses on a task, for example “old” versus “new” responses on recognition memory tasks, are thought to rely on distinct cognitive processes (Wixted, 2007; Yonelinas, 1994). As prior work with the sentence verification task suggests semantic distance differentially affects TRUE and FALSE responses for both EEG and RTs (Beltrán et al., 2018; Carpenter & Just, 1975; Fischler et al., 1984; Wason, 1961), we analyzed the TRUE and FALSE trials separately.

From 8,117 artifact-free property epochs, we first excluded 946 trials with incorrect responses (accuracy rate of 88.35%). We then selected the epochs for which relevant scores were available as described earlier (428 unique concept—property pairs, 6,414 trials). We additionally removed 5 trials with response times greater than 2,600 ms, which was the time allotted for responding on each trial. For the artifact-free, correct trials with relevant scores available, we conducted separate single-trial analyses for TRUE (*n* = 3,126) and FALSE (*n* = 3,283) trials.

### Modeling Approach

**Figure 2.**
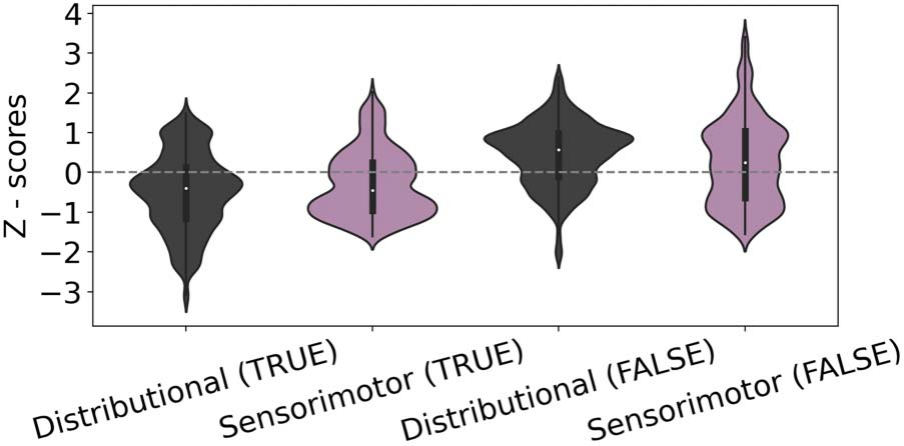
Violin plots of distributional and sensorimotor cosine distance measurements. Scaled scores are presented for both TRUE and FALSE property trials. *Z* = 0 is marked with a dashed horizontal line.

For both RTs and EEG data, we constructed four competing linear mixed effects regression (LMER) models to account for the high subject- and item-level variance, and evaluated their relative fit using the Akaike Information Criterion (AIC) scores. EEG models predicted the mean amplitude of EEG voltage, and RT models predicted the mean RT. As noted above, models for TRUE and FALSE property trials were constructed separately. To increase the interpretability of the beta weights, all predictors were scaled by z-scoring across the entire set of trials (i.e., both TRUE and FALSE). Figure 2 shows the distributions of the scaled measurements of distributional distance and sensorimotor distance in each sort of trial. All models were fitted using the lme4 package in R (Bates et al., 2015).

For the EEG data, we first measured the mean EEG voltage at each electrode in each of the seven 100 ms windows starting from 0–100 ms, i.e., 100 ms after the onset of the property word up to 600–700 ms for each single-trial EEG epoch. Further, we captured the location information of each scalp channel using three dimensions: the horizontal (left/right) *X* axis, vertical (anterior/posterior) *Y* axis, and the *Z* axis that informed on depth (top of the head to periphery). The predictors *X*, *Y*, and *Z* thus represent continuous variables corresponding to three dimensions along which the electrodes are distributed (for details, see Section 1 of Supplementary Materials). Predictor *X*, then, encodes the *X*-axis location values for all 29 electrodes, and *Y*, and *Z* encode the corresponding values along their respective axes. We allowed these scalp topography dimensions to interact with our predictors of interest (Winsler et al., 2018). Conceptualizing EEG channels as either random or as different levels of a fixed effect poses challenges due to correlations between adjacent channels (see Kappenman & Luck, 2012 for discussion). Rather than including an explicit Electrode factor, our approach treats observations at each electrode as signals recorded at a particular set of *X*, *Y*, and *Z* coordinates, allowing us to model the similarity of signals from nearby locations (Winsler et al., 2018; see Tremblay & Newman, 2015 for a related approach). By modeling each predictor’s interactions with scalp dimensions, this approach has the added advantage of enhancing the interpretability of topographic effects.

#### Models.

The four competing models we employ are: the Base model, the Distributional model **D**, the Sensorimotor model **S**, and the combined or Distributional and Sensorimotor model **DS**. The Base model included the logarithmic word frequency measurement of the property word (e.g., “red”) as the only predictor. For the three subsequent models (**D**, **S**, and **DS**), word frequency was included as a control predictor.

The description for the Base model of RTs is:

$$\text{RT}=\text{Intercept}+\text{word}\_\text{frequency}+\left(1|\text{subject}\right)+\left(1|\text{word}\right)$$

And for the EEG data, it is:

$$\begin{array}{l} \text{Voltage}=\text{Intercept}+X+Y+Z+\text{word}\_\text{frequency}\\ \;+\text{word}\_\text{frequency}:X+\text{word}\_\text{frequency}:Y+\text{word}\_\text{frequency}:Z\\ \;+\left(1|\text{subject}\right)+\left(1|\text{word}\right) \end{array}$$

The second model was the Distributional model (referred as ‘**D**’), which included the distributional distance measured between the concept—property pairs (e.g., “APPLE” – “red”) as a predictor along with word frequency.

The description for the Distributional model for RTs is:

$$\text{RT}=\text{Intercept}+\text{word}\_\text{frequency}+\text{distributional}\_\text{distance}+\left(1|\text{subject}\right)+\left(1|\text{word}\right)$$

And for the EEG data:

$$\begin{array}{l} \text{Voltage}=\text{Intercept}+X+Y+Z+\text{word}\_\text{frequency}+\text{distributional}\_\text{distance}\\ \;+\text{word}\_\text{frequency}:X+\text{word}\_\text{frequency}:Y+\text{word}\_\text{frequency}:Z\\ \;+\text{distributional}\_\text{distance}:X+\text{distributional}\_\text{distance}:Y\\ \;+\text{distributional}\_\text{distance}:Z+\left(1|\text{subject}\right)+\left(1|\text{word}\right) \end{array}$$

Similarly, the Sensorimotor model **S** included the sensorimotor distance with word frequency, and the Distributional and Sensorimotor model **DS** included both the distance measures, along with word frequency. With four models for each of the seven 100 ms windows, there were a total of 28 EEG models for the TRUE trials and 28 for the FALSE trials. Likewise, there were four models of RTs for the TRUE trials and four for the FALSE ones. The code for running these models for both RT and EEG data are available in the OSF repository that can be accessed using the link http://osf.io/6efp8.

#### Model Comparison.

As previously mentioned, we use the Akaike Information Criterion (AIC) scores for model comparison since not all our models are nested (e.g., models with only distributional or only sensorimotor distance measures). AIC scores are measured as (−2 ln(*L*) + 2*k*) where *L* is the likelihood of the model and *k* is the number of parameters, and thus penalizes models for complexity (Burnham & Anderson, 2004). Importantly, AIC values are not evaluated against a distribution under a specific null hypothesis. They are used to compare the relative fit of models to the same dataset, and so are meaningful only in comparison to other candidate models of the same data. For this reason, AIC scores are suitable to evaluate model fit in each time window and were used to compare the relative performance of the **D**, **S**, and **DS** models across the epoch. An AIC difference of 0–2 is typically interpreted as showing that the models are essentially indistinguishable in terms of their support by the data. A difference between 4–7 suggests the model with lower AIC provides a more likely account of the data, and a difference of greater than 10 suggests that the model with higher AIC has substantially less support, indicating a robust improvement (Burnham & Anderson, 2004). We present AIC comparisons using these criteria, and take a reduction of AIC by 10 or more as an indication for a robust effect.

On a related note, as both the distributional and sensorimotor cosine distance measures were weakly positively correlated (Pearson’s correlation, TRUE trials 0.28, *p* < .001, and FALSE trials 0.18, *p* = 0.007), and word frequency had a weak negative correlation with distributional distance within FALSE trials (Pearson’s *r* = −0.18, *p* = 0.007), we calculated the variance inflation factor (VIF) using the CAR package in R to detect potential multicollinearity (Fox & Weisberg, 2019). For the biggest model, Distributional + Sensorimotor (DS) that included all of the predictors, the VIF values for all fixed effects including interactions ranged from 1.03 to 1.76 across both the TRUE and FALSE trials. Typically, a VIF value exceeding 4 warrants investigation of the model structure, and when it exceeds 10, it is considered to be a sign of multicollinearity that needs correction (Fox & Weisberg, 2019). Our biggest models’ VIFs around 1 indicate that multicollinearity was not a concern in these models and all the predictors could be safely included together in the models.

#### Scalp Distributions.

Interactions of each predictor with scalp topography variables informed whether and how the effect varied across channels arrayed in these three spatial dimensions. For example, in the Base model structure, the interaction of word frequency with the *X*-axis indicated how its effect changed from the left hemisphere channels to the right hemisphere channels. Similarly, the *Y*-axis interaction referred to changes from the posterior to anterior channels, and the *Z*-axis referred to changes from central channels at the top of the head to peripheral channels closer to the ears.

We used these beta values from interactions between scalp topography variables and our predictors from the combined model to estimate the topographic distribution of their effects (Emmorey et al., 2020; Winsler et al., 2018, 2023). By building models with three scalp dimensions instead of modeling EEG at each individual channel, beta estimates were more schematic than the original recordings. However, these scalp distributions comprised stable and reliable synopses of the topographic effects of each predictor (see Smith & Kutas, 2015 for discussion).

## RESULTS

In this section, we begin by describing RT and EEG comparison between the TRUE and FALSE trials, as these trials were modeled separately. Next, we look at the performance of the Distributional model D, Sensorimotor model S, and the combined model DS in each kind of trial, first for the RTs and then for the EEG data. The EEG model comparison is followed by the final subsection of the results, where we report the topographic profiles of our distributional and sensorimotor distance predictors.

### RTs TRUE vs. FALSE

TRUE trials had a mean response time of 952 ms (*SD* = 339), while FALSE trials had a mean response time of 945 ms (*SD* = 322). We compared the RTs from all trials using a linear mixed-effects model with a fixed effect of trial type (i.e., True vs. False) and random effects of subjects and words. Here (as elsewhere) p-values were estimated using Satterthwaite’s degrees of freedom approximation as implemented in the lmerTest package (Kuznetsova et al., 2017), and it suggested no significant response time differences between the two kinds of trials (*p* = 0.59).

### RT Model Comparison

**Figure 3.**
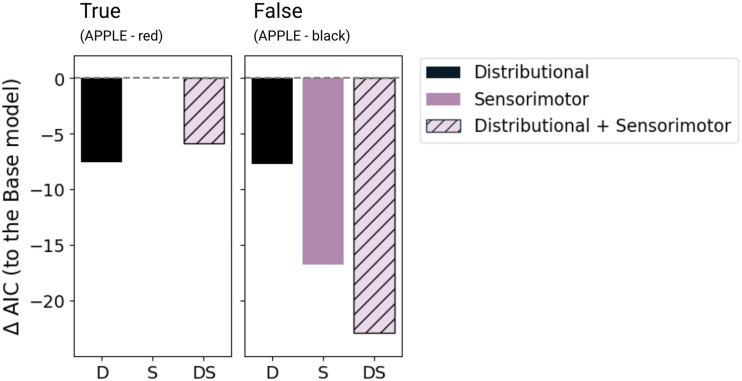
AIC comparison of RT models. AIC is scaled by subtracting the Base (word frequency) model’s AIC from each of the three models of interest, such that 0 scaled AIC suggests no improvement on the Base model. The lower the scaled AIC value, the better the model fits.

We compared the four models we constructed using the AIC measurements. The AIC of each theoretical model of interest (i.e., Distributional D, Sensorimotor S, Distributional + Sensorimotor DS) was scaled by subtracting the AIC of the Base B model that only included word frequency as a predictor. Figure 3 presents AIC values of D, S, and DS models scaled to the Base model of RTs.

For the TRUE trials, the distributional distance predictor model D yielded an improvement in model fit relative to the Base model that included only word frequency as the sole predictor (ΔAIC to Base = −7), but it did not cross the robustness criterion. No improvements from the Base model fit were offered by the Sensorimotor distance model (ΔAIC to Base = 0). In contrast, for the FALSE trials RTs, the Sensorimotor distance model S robustly lowered the AIC relative to the Base model (ΔAIC to Base = −16). The combined model DS further reduced AIC by 5 compared to S (ΔAIC to S = −5, and ΔAIC to Base = −21), indicating that DS provided a more likely account of the data, though this difference did not meet the robustness criterion.

**Figure 4.**
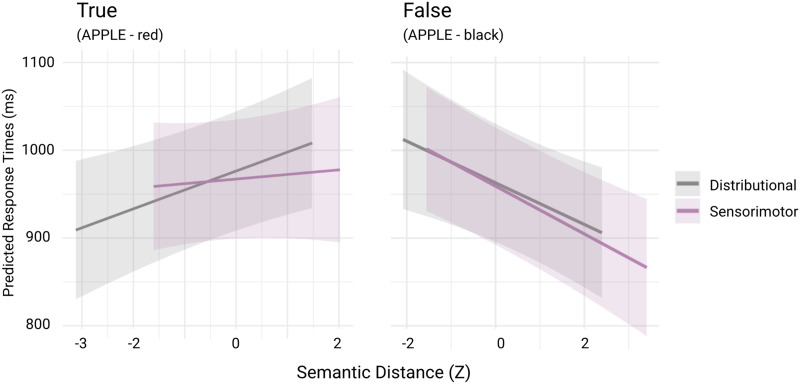
Marginal effects of Distributional and Sensorimotor predictors on RTs from the combined DS model. The lines represent model-derived predictions and the shaded area shows the 95% confidence intervals, shown separately for TRUE and FALSE trials.

To compare the direction of effects of each of our predictors on RTs in the TRUE and the FALSE trials, we present beta estimates from the combined model DS for both types of trials in Table 1 (VIF values ranged from 1.00 to 1.09), with predicted response times plotted in Figure 4, to allow us to compare the relative importance of our three predictors while controlling for differences in the others. Similar estimates from each of the RT models, including the Base, Distributional, and Sensorimotor, are presented in Section 2 of the Supplementary Materials.

**Table 1.** RTs DS model beta estimates. The significant predictor *p*-values are highlighted in **bold** fonts. The estimates suggest that for TRUE trials, word frequency and distributional distance each made a significant contribution to RT variance; an increase in word frequency reduced the RTs while an increase in distributional distance increased the RTs. For the FALSE trials, increasing both distributional and sensorimotor distance significantly reduced the RTs.

**Predictors**	**TRUE**			**FALSE**		
	**Estimates**	**Std. error**	***p*-values**	**Estimates**	**Std. error**	***p*-values**
Intercept	983.37	34.56	**<.001**	971.73	34.73	**<.001**
Word Frequency	−35.26	9.72	**<.001**	−7.67	8.69	.381
Distributional	21.58	7.97	**.006**	−23.73	8.62	**.006**
Sensorimotor	5.24	10.64	.6	−27.25	6.57	**<.001**

The estimates suggest that for TRUE trials, there was a significant *decrease* of 35 ms in response time with each standard deviation increase in the word frequency (*p* < .001) and a 22 ms *increase* in the response time with each standard deviation increase in distributional distance (*p* = 0.006). For the FALSE trials, on the other hand, we see no significant effect of word frequency, a significant *decrease* of 24 ms in response time as distributional distance increases (*p* = 0.006) and a decrease of 27 ms as sensorimotor distance increases (*p* < .001). The opposing effects of distributional distance on TRUE and FALSE trials thus supports our decision to analyze them separately.

### ERPs

**Figure 5.**
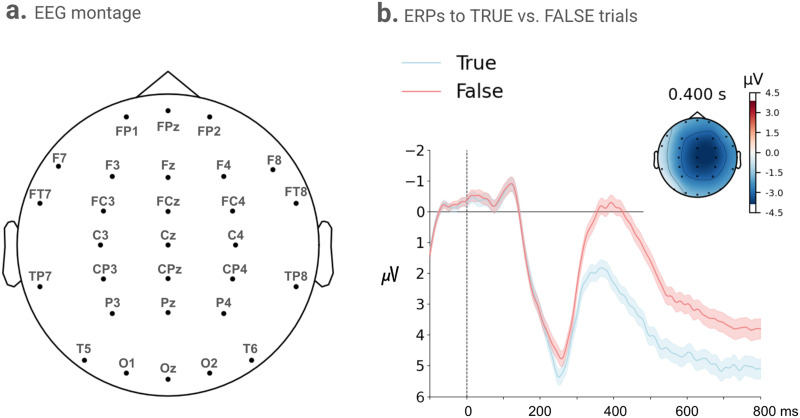
(a) EEG Montage. Electrode layout of the EEG, each dot represents an electrode position with channel names; Cz is the centre with coordinates [0, 0, 0] and the scalp contour has a 10 cm radius. (b) ERPs averaged from all scalp electrodes to TRUE vs. FALSE trials with 95% confidence interval. Negative voltage is plotted upwards. The topographic distribution of the difference wave for ERPs to FALSE minus TRUE trials is shown at 400 ms. Its blue color reflects larger negative ERPs to FALSE trials relative to TRUE ones.

For the EEG data, we show the trial averaged Event-Related Potentials (ERPs) for TRUE and FALSE trials in Figure 5b. The topography of the difference wave between the two ERPs (TRUE subtracted from FALSE) at 400 ms shows a larger negativity for FALSE trials that was prominent over the centroparietal electrodes.

### EEG Model Comparisons

**Figure 6.**
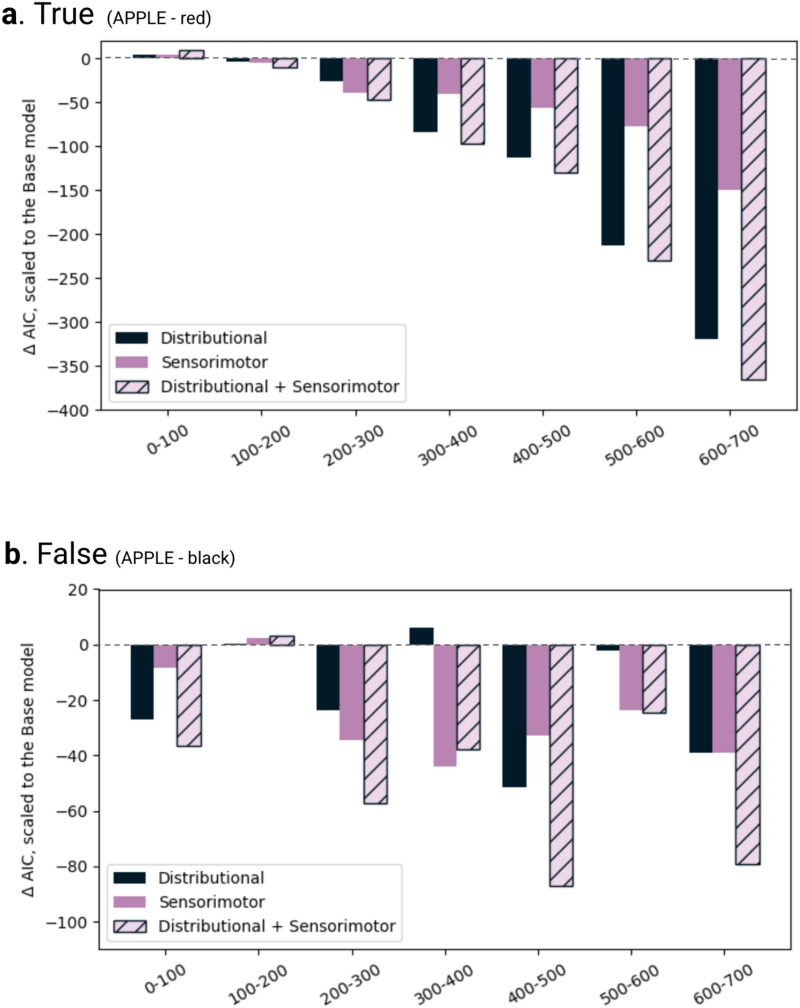
AIC comparison of EEG models AIC is scaled by subtracting the Base model’s AIC similar to the RTs models (Figure 3). Panel (a) shows the model comparison for the TRUE trials and (b) for the FALSE trials. The *X*-axis of both subplots marks each successive 100 ms time window. Note that ΔAIC range on the *Y* axis for Figure 6a and 6b are different as the relative EEG fits varied across the TRUE and FALSE trials.

Figure 6 compares the AIC values for the EEG data; all three models of interest—D, S, and DS—are scaled to the Base model for the visualization purpose. This section reports the AIC differences between the EEG models, with a reduction of 10 or more taken as an indication of robust model fit improvement.

#### 0–100 ms.

For the TRUE trials, AIC comparison (Figure 6a) showed that none of the models provided a better fit than the Base model that included only word frequency. For the FALSE trials (Figure 6b), the distributional model D improved the fit over the Base model robustly (ΔAIC to Base = −26). The combined model DS further offered a more likely account compared to D (ΔAIC to D = −7) but did not reach the robustness criterion.

#### 100–200 ms.

For the TRUE trials, neither the Distributional distance D (ΔAIC to Base = −3), the Sensorimotor distance S (ΔAIC to Base = −5), nor the combined DS (ΔAIC to Base = −9) model robustly improved on the Base model, though S and DS offered a more likely fit. For the FALSE trials, the models of interest provided a poorer fit than the Base model, with the Distributional D (ΔAIC to Base = 0), the Sensorimotor S (ΔAIC to Base = 2), and the combined DS (ΔAIC to Base = 3) fits leading to higher AIC values.

#### 200–300 ms.

For the TRUE trials, the Distributional distance model D (ΔAIC to Base = −25) and the Sensorimotor distance model S (ΔAIC to Base = −38) both improved the model fit over the Base model robustly, with the S model performing better than the D model (ΔAIC to D = −13). The combined model DS further lowered the AIC (ΔAIC to S = −7), suggesting the DS model was more likely than S, albeit not a ‘robust’ improvement.

#### 300–400 ms.

For the TRUE trials, the Distributional distance model D (ΔAIC to Base = −84) and Sensorimotor distance model S (ΔAIC to Base = −40) robustly improved the fit over the Base model, with D performing better (ΔAIC to S = −44), and the combined model DS provided a robustly improved fit compared to D (ΔAIC to D = −12). For the FALSE trials, the Sensorimotor distance model S offered an improvement over the Base model (ΔAIC to Base = −44). The combined model DS further lowered the AIC relative to the S model (ΔAIC to S = −6), suggesting an improvement that did not reach the robustness criterion.

#### 400–500 ms.

For TRUE trials, the Distributional distance model D (ΔAIC to Base = −112) and Sensorimotor distance model S (ΔAIC to Base = −56) both improved the fit over the Base model, with D performing better than S (ΔAIC to S = −56). The combined model DS further lowered the AIC compared to the D model (ΔAIC to D = −17). Similar trend was observed in the FALSE trials, where both the Distributional model D (ΔAIC to Base = −51) and Sensorimotor model S (ΔAIC to Base = −32) improved the fit over Base, and the DS model provided a robust improvement on the D model (ΔAIC to D = −35).

#### 500–600 ms.

In this window, for the TRUE trials, both the Distributional distance model D (ΔAIC to Base = −212) and the Sensorimotor distance model S (ΔAIC to Base = −77) improved the fit over Base, with a better fit offered by the D model (ΔAIC to S = −135). The combined model DS further lowered the AIC compared to the D model (ΔAIC to D = −18). For the FALSE trials, the Sensorimotor model S improved over the Base model (ΔAIC to Base = −23), however the Distributional distance model D did not (ΔAIC to Base = −2). The combined DS model offered no improvement over S (ΔAIC to S = −0.8).

#### 600–700 ms.

For the TRUE trials, the Distributional model D (ΔAIC to Base = −319) as well as the Sensorimotor model S (ΔAIC to Base = −149) improved the fit over the Base model, with D performing better than the S model (ΔAIC to S = −169). The combined model DS further robustly improved the fit compared to D (ΔAIC to D = −46). For the FALSE trials, the model comparison suggested the Distributional model D and the Sensorimotor model S each yielded similar improvement to the Base model (ΔAIC to Base = −38), and the combined model robustly improved the fit relative to both D and S models (ΔAIC to D and S both = −40).

### Scalp Topographies of the Effect

**Figure 7.**
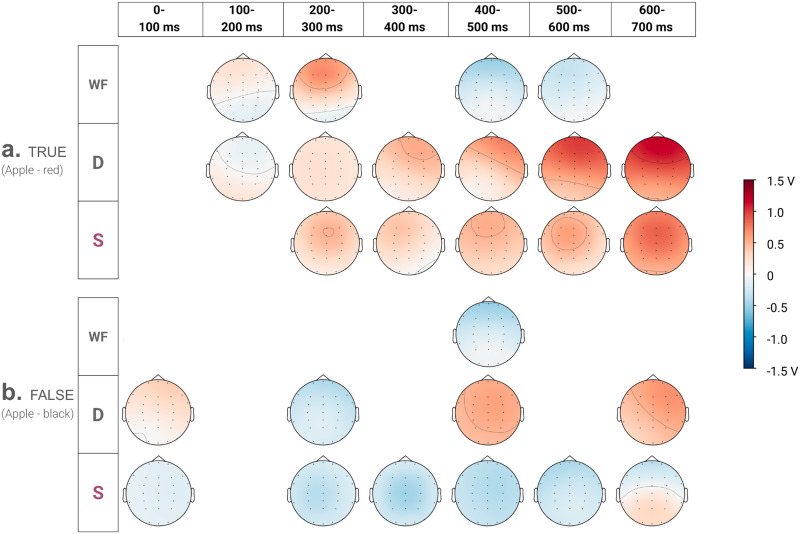
Topographic distribution of beta values from the combined model (DS) of EEG data. Estimate *β*_*i*_ for a channel (i) is estimated as *β* + *β*_*x*_ · *X*_*i*_ + *β*_*y*_ · *Y*_*i*_ + *β*_*z*_ · *Z*_*i*_, where *β* is the coefficient of the main effect for a predictor, *β*_*x*_ the coefficient of the interaction of the predictor with the topography axis *X* (similar for *β*_*y*_, and *β*_*Z*_) and *X*_*i*_, *Y*_*i*_, *Z*_*i*_ are the coordinates of channel *i*. The detailed explanation of the estimations are provided in Section 3 of the Supplementary Materials. Only scalp distributions corresponding to significant effects reported in Table 2 are shown.

Table 2 reports the significant effects for all predictors in the combined DS model after correcting for multiple comparisons. The correction was done by collecting all estimates produced by the combined DS model across the seven windows and using a false discovery rate (FDR) procedure that assumes no dependency structure among tests (Benjamini & Yekutieli, 2001). We chose the DS model with all predictors so we could present coefficients under the condition where each predictor is simultaneously competing with a constant set of predictors in the model. The DS model was also overall the most likely model across the majority of the time windows and for both TRUE and FALSE trials. Along with reporting the coefficients of the significant predictors, we present estimated scalp distributions of their effects in Figure 7 using the coefficients from the DS model. Furthermore, to illustrate the consistency in the direction of predictor effects across models, we present an analogous figure to Figure 7 in Section 4 of the Supplementary Materials that shows beta estimates from the best-fitting models (instead of the combined model DS) for each analysis window.

**Table 2.** EEG DS model significant beta estimates. Each column presents the FDR-corrected significant regression coefficients (*p* < .05) for Word Frequency (WF), Sensorimotor distance (S), and Distributional distance (D) predictors from the DS model of EEG data from TRUE trials (left) and FALSE trials (right). For each predictor, the corresponding regression coefficient (*β*, in *μ*V) for main effects as well as for interactions with specific axes are listed.

**Time (ms)**	**TRUE**			**FALSE**		
	**WF**	**D**	**S**	**WF**	**D**	**S**
**0–100**					0.17, 1.79 (Y)	−0.12
**100–200**	1.97 (Y)	−1.50 (Y)				
**200–300**	4.45 (Y)	0.18	0.25		−0.31	−0.23
**300–400**		0.32, 2.32 (Y)	1.87 (Y)			−0.25, −2.44 (Z)
**400–500**	−2.64 (Y)	0.39, 3.30 (Y)	0.38	−1.85 (Y)	0.49	−0.33
**500–600**	−1.71 (Y)	0.68, 3.57 (Y)	0.33			−0.31
**600–700**		0.84, 3.87 (Y)	0.61		0.47	−3.69 (Y)

#### TRUE Trials.

Figure 7a shows the estimated scalp distributions, and reveals the anterior positivity from 100 to 300 ms and negativity from 400–600 ms as word frequency increases. Word frequency significantly interacted with the *Y* axis in 100–300 ms with a positive estimate suggesting that high-frequency words elicited more positive ERPs over anterior compared to posterior electrode sites (Table 2). Similarly, the later effects of word frequency, in 400–600 ms, reflected more negative ERPs over anterior sites as word frequency increased.

Beginning 100 ms post-onset until the end of the epoch, distributional distance significantly interacted with the *Y* axis in all windows except the 200–300 ms window, as the distributional distance increased, property terms elicited larger anterior positivities (Figure 7a and Table 2). Sensorimotor distance exhibited a significant main effect in five time windows from 200–700 ms (Table 2), and an interaction with the *Y* axis reflecting more positive ERPs in anterior sites in 300–400 ms. As shown in Figure 7a, sensorimotor distance elicited a similar anterior positivity to that for distributional distance.

#### FALSE Trials.

In the 0–100 ms window, both the distributional and sensorimotor distances had a main effect, and distributional distance also interacted with the *Y* axis (Table 2). Figure 7b shows that more positive ERPs were correlated with greater distributional distance, especially over anterior sites in this interval, while sensorimotor distance was generally correlated with more negative ERPs.

Distributional distance also had a significant main effect 200–300 ms, 400–500 ms and 600–700 ms (Table 2), with more negativity in 200–300 ms and then larger positivities in the later time windows (Figure 7b). Sensorimotor distance exhibited main effects from 200 ms until the end of the epoch. It also showed significant interaction with the *Z* axis in 300–400 ms and with *Y* axis in 600–700 ms. The main effects and the *Z* interaction, until 600 ms reflect the association between greater sensorimotor distance and more negative ERPs in general, while in the last 600–700 ms window greater sensorimotor distance was correlated with larger posterior positivities in the EEG.

## DISCUSSION

Hybrid models of semantic memory have attempted to reconcile the earlier division between distributional and grounded approaches, suggesting that both contribute to semantic knowledge (Barsalou et al., 2008; Davis & Yee, 2021). Motivated by these proposals, the present study asked whether distributional, sensorimotor, or both types of information influence behavior and neural activity during conceptual processing. To do this, we operationalized distributional information by using GloVe embeddings to calculate cosine distance measures between concept—property pairs from a property verification task; likewise, we operationalized sensorimotor information as cosine distance measures using vectors derived from the Lancaster Sensorimotor Norms. We then modeled response times (RTs) and single-trial EEG elicited by property words. Our results are overall consistent with the *hybrid* models’ prediction showing that both distributional and sensorimotor measurements reliably and uniquely predict variance in the EEG as well as in RTs.

Importantly, inferences regarding the recruitment of experiential versus distributional information depend fundamentally on the construct validity of our operationalizations of these measures. In particular, the distributional measure might reflect varied semantic knowledge, including knowledge of sensorimotor experience. Thus, the ability of a distributionally-derived measure to predict human behavior (or neural responses) does not entail that the representations guiding behavior are distributional in nature—at the same time, it serves as a *proof of concept* that such representations can be gleaned from language alone. Further, we note that the present study utilized well-validated computational proxies for distributional statistics and the sensorimotor information via the GloVe model (Ettinger & Linzen, 2016; Reilly et al., 2023), and the Lancaster Sensorimotor Norms (Lynott et al., 2020; Wingfield & Connell, 2023), respectively.

We begin below by considering the implications of our finding for hybrid models of semantic memory, arguing that our results suggest distributional and grounded information sources make independent contributions to semantic processing. We go on to consider the implications of differences in effects on the TRUE versus FALSE trials, arguing that they highlight the context-sensitivity of conceptual processing. Next we explore the dynamic nature of distributional and sensorimotor activations in the neural data, finding only limited support for suggestions regarding the temporal precedence of distributional information. Overall, our data suggest semantic memory representations are sensitive to both embodied sources and information from the statistics of language, and that they each contribute differentially according to contextual demands.

### Recruitment of Both Distributional and Sensorimotor Resources

Our findings suggest that participants recruit both distributional and sensorimotor information to perform the property verification task, supporting the relevance of both language and experiential resources in this conceptual task. RT model estimates for TRUE trials show that participants responded slower as distributional distance increased, and reveal no significant effects of sensorimotor distance (see Table 1 and Figure 4). For example, when the distributional distance between the concept and the property was relatively large (as in “YAMS” – “firm”) participants took longer to respond than when the relevant concepts were closer (as in “ZEBRAS” – “striped”). Slower responses to increased distributional distance align with previous findings that show GloVe embeddings serve as a good computational proxy for semantic relatedness encoded via language statistics (Auguste et al., 2017; Ettinger & Linzen, 2016; Reilly et al., 2023). Behavioral data thus suggest property verification on the TRUE trials was supported primarily by word co-occurence.

For the FALSE trials, although the AIC difference between the DS and S models did not meet the threshold for robust support, the DS model was nonetheless considerably more likely. The DS model estimates show that both predictors showed effects, with the sensorimotor distance exhibiting a slightly larger effect size (see Table 1). Responding that a property did not hold for a concept was easier when the sensorimotor distance between them was high (as in “THUMBS” – “moan” in which the concept has a tactile hand profile and the property an auditory mouth one) than when it was low (“HEADLIGHTS” – “opaque” in which both words are known visually). That is, participants were able to settle on the FALSE response more quickly when the two words in the trial were more distinct in their sensorimotor profiles. Like sensorimotor distance, distributional distance was also negatively correlated with response time for FALSE trials; this was in contrast to the effect of distributional distance for TRUE trials (in which increases in distributional distance were positively correlated with response time); see Figure 4 for DS model fits, and Section 2 in the Supplementary Materials to observe direction of fits when the Distributional and Sensorimotor predictors are fitted independently of each other.

In sum, behavioral data suggest responses to TRUE trials were supported by word-frequency and distributional information, while responses to FALSE trials were supported by both sensorimotor and distributional information. The EEG data also revealed that both distributional and sensorimotor distance influenced the brain response, albeit with differing effects in TRUE and FALSE trials. Notably, the EEG topographic profiles of distributional and sensorimotor distance effects were broadly similar to each other between 200–400 ms, both eliciting small anterior positivities in TRUE trials (see Figure 7a) and small negativities in FALSE trials (see Figure 7b). A plausible reading of this finding is that distributional and sensorimotor information may tap overlapping neural processes during the early stages of semantic retrieval 200–400 ms post word onset, although the apparent similarity in the two effects may stem from the sparsity of our electrode montage (see Figure 2), or the limited spatial resolution of EEG.

However, the present study did reveal differences in the EEG topographies to distributional and sensorimotor information in the latter half of the epoch. Clear differences in the two effects emerge 400 ms after word onset, as distributional distance in FALSE trials was associated with larger positivities similar to the distributional effects in the TRUE trials, while the sensorimotor distance predictor continued to elicit more negative ERPs (see Figure 7b and Section 4 of the Supplementary Materials). Dissociable effects of distributional and sensorimotor distance point to different configurations of brain areas supporting the recruitment of each sort of information. These results are consistent with fMRI studies that suggest conceptual processing tasks recruit sensorimotor brain regions relevant for embodied approaches to meaning as well as transmodal regions that are sensory-independent (Bi, 2021; Davis & Yee, 2021; Wang et al., 2020). However, the present study is also limited by the spatial resolution of EEG. Future research would benefit from the use of methods such magnetoencephalography and electrocorticography with good resolution in both spatial and temporal dimensions.

### Context-Sensitivity of Information Recruitment

Beyond showing that participants used both distributional and sensorimotor information to perform the property verification task, the present study suggests that task demands impact the extent to which each sort of information is recruited along with the timing of its recruitment. Sensorimotor distance, for example, explained response time variance beyond both word frequency and distributional distance in the FALSE trials. In TRUE trials, however, it did not explain additional variance above word frequency. Our EEG models likewise appear to suggest these predictors’ explanatory potential varied across time during word processing, and differs in TRUE and FALSE trials. For the TRUE trials, in the 200–300 ms window, the DS model was more likely than the second best-performing Sensorimotor model S, though this difference did not reach the robustness criterion. Beginning 300–400 ms, the Distributional model D consistently outperformed the Sensorimotor model S, though the combined DS model provided the best account of the data from 300–400 ms until the end of the epoch. For the FALSE trials, however, the pattern varied across the seven 100 ms windows, showing a more dynamic interplay between sensorimotor and distributional information when faced with an unusual sequence of words (e.g., “APPLE” – “black”). These variations may reflect differences due to task-specific processes such as re-evaluation or conflict resolution triggered by encountering a mismatched property term in the FALSE trials versus a facilitated integration in the TRUE trials (Beltrán et al., 2018; Fischler et al., 1984).

Moreover, observed differences in EEG effects for TRUE and FALSE trials are largely in line with the literature on grounded meaning. First, the importance of distributional distance for EEG to TRUE trials (Figure 6a) is reminiscent of prior studies highlighting a strong role for linguistic co-occurrence in semantic processing when the context supports the upcoming word (Connell, 2019). Second, for the FALSE trials, we note the performance of the Sensorimotor model S being equal to or better than that of the Distributional model D in three out of the four windows after 300 ms (Figure 6b). Earlier research suggests sensorimotor information may be recruited later for combined concepts, such as BLACK APPLE, that are not supported by real-world experience. For example, ERP effects of modality switching are later for FALSE trials than for TRUE ones (Hald et al., 2013) as well as for novel compounds (Connell & Lynott, 2011). This sensorimotor recruitment is also consistent with the idea that greater sensorimotor support is required to combine concepts on FALSE trials, as neuroimaging research suggests there is more activation in modality-specific brain regions when participants judge less accessible attributes (Hoenig et al., 2008).

The variability in the relevance of sensorimotor information for affirming versus rejecting the applicability of a property term is especially compatible with the COntext-Dependent Embodied Simulation (CODES) model that suggests grounded resources are flexibly recruited as a function of their utility for the current task. Sensorimotor recruitment is highly variable, being most important in situations that require deep semantic processing and inferential elaboration (Winkielman et al., 2018, 2023). Our results also align with the Linguistic Shortcut Hypothesis and its suggestion that the recruitment of sensorimotor resources is not automatic and is more likely in contexts that promote deep processing (Connell, 2019). The importance of sensorimotor information in the present study is likely related to our use of the property verification task, a semantic processing task that benefits from perceptual simulation. Moreover, we also included associated false trials, a manipulation known to increase sensorimotor engagement (Kan et al., 2003). Sensorimotor distance effects on the brain response might be weaker (or even absent altogether) with different task contexts.

### Dynamics of Distributional Distance Effects

One goal of the present study was to examine the precise timing of the recruitment of distributional versus experientially grounded information in conceptual processing. LASS suggests that word recognition triggers the immediate engagement of both linguistic and grounded semantic representations, but the earliest stages involve the generation of word-associates (e.g., “pie”, “red” or “crispy” for “apple”) as pointers to more detailed semantic information (Barsalou et al., 2008). The way we operationalize distributional information in the current study differs somewhat from that posited in LASS in which the linguistic system engages with semantic processing through word-form associations and semantic heuristics. By contrast, more recent hybrid accounts draw from distributional semantics, an approach that suggests semantic information is encoded in structures developed via language statistics (Connell, 2019; Winkielman et al., 2023). Using cosine distances for vectors built from the GloVe model (Ettinger & Linzen, 2016; Pennington et al., 2014; Reilly et al., 2023), our distributional measure captures both the associative relations between words as posited in the LASS model in addition to semantic information encoded via more sophisticated language statistics.

Work on visual word processing suggests ERP effects in the initial 200 ms are primarily sensitive to the complexity of the visual word form, with orthographic and lexical effects emerging around 200 ms, and semantic effects after 300 ms (Dufau et al., 2015). Language-related factors known to influence the initial (viz., 200 ms) stages of the ERP response to words are word length and word frequency (Hauk & Pulvermüller, 2004). In keeping with prior reports of ERP frequency effects emerging 150–190 ms (Hauk & Pulvermüller, 2004), the Base model—with word frequency as its sole predictor—provided the most robust fit to our EEG data in the 100–200 ms interval for both the TRUE and the FALSE trials.

By contrast, in the 0–100 ms window, the Base model accounted for most of the EEG variance on the TRUE trials. For the FALSE trials, the Distributional model D robustly improved upon the Base model, whereas the DS model’s additional improvement did not meet the robustness criterion, suggesting a strong early influence of distributional distance. This early effect of distributional distance thus provided support for models such as LASS that argue for the importance of linguistic factors in early stages of processing. However, as these effects were found only for words in the FALSE trials (e.g., “APPLE” – “black”), it suggests the word association strategy is more likely to be recruited when the incoming word violates expectations. To date, reports of language context effects on ERPs to words have primarily emerged after 250 ms (see Kutas & Federmeier, 2011 for a review), though effects around 200 ms (e.g., Yan & Jaeger, 2020; Yang & Perfetti, 2006) and even as early as 120 ms (Penolazzi et al., 2007) have been observed. As ERP context effects have not previously been reported between 0–100 ms, this finding is to be interpreted with caution.

We also found distributional distance to be an important predictor of the brain response throughout the epoch, in keeping with models suggesting linguistic and grounded representations are interdependent (Louwerse, 2011). Our finding that distributional distance predicts neural and behavioral responses independent of sensorimotor representations substantiates distributional embeddings as a distinct class of information that offer unique semantic contributions, and are not simply a shallow resource utilized because it is computationally cheaper (Connell, 2019; Ettinger & Linzen, 2016; Reilly et al., 2023; Wang et al., 2020).

### Dynamics of Sensorimotor Distance Effects

Grounded theories of concept representation suggest that sensorimotor and affective representations are reactivated in the course of concept retrieval (Barsalou et al., 2008). The dimensions of experiential knowledge captured by the Lancaster sensorimotor norms are presumed to index neural processing systems for vision, touch, audition, olfaction, taste, and interoception, as well as those relevant for motor control. Neural activation associated with sensorimotor distance may reflect the reactivation of associated experiential information in the sensorimotor cortex. Perhaps more likely, however, it may be related to activity in the default mode network thought to act as convergence zones for the concurrent reactivation of sensory-motor information. Activity in these heteromodal regions scales with the number of sensory-motor features relevant for word meaning, a result that has been argued to suggest these regions encode multimodal combinations of sensory and motor features (Fernandino et al., 2016).

Although the behavioral data indicated no support for the recruitment of sensorimotor information in the TRUE trials, the EEG data suggested that both distributional and sensorimotor distance influenced the brain response, albeit with differing effects in TRUE and FALSE trials. Notably, for the TRUE trials, the 200–300 ms window marks the point at which the Sensorimotor model began to offer better fit than the Base model (see Figure 6a), and the sensorimotor predictor continued to account for unique variance in subsequent intervals where the DS model was best fitting.

The 200 ms onset of sensorimotor distance effects in TRUE trials aligns with that reported in other EEG studies of grounded meaning (see, e.g., Bardolph & Coulson, 2014). For example, Pulvermüller et al. (2001) observed differential activations in frontocentral EEG around 200 ms due to different kinds of action words—such as leg-related (e.g., walk) or face-related (e.g., talk) words—indicating differences in activity for words based on their motor associations. Results of the present study are thus in line with prior ERP studies that show the sensory and motor associations of individual words contribute considerably to neural representations activated around the 200–400 ms time (Kiefer et al., 2007; Pulvermüller et al., 2001, also see, Hauk et al., 2004 and Pulvermüller, 2005).

Further, given that sensorimotor distance was more relevant for judging FALSE trials, the difference in sensorimotor measure topography also lines up with the observations of functional neuroimaging studies that show sensorimotor experience based information measurements explain neural activations across different levels of abstraction, in high-level transmodal regions as well as in sensorimotor cortical regions (Fernandino et al., 2022; Kuhnke et al., 2020). The scalp variations for the sensorimotor predictor post 400 ms are consistent with the claim that the representation of grounded knowledge in semantic memory involves different levels of information abstraction that is accessed efficiently based on contextual demands (Binder & Desai, 2011; Connell & Lynott, 2013; Winkielman et al., 2023).

## CONCLUSION

The hybrid accounts, while acknowledging the contribution of both distributional and sensorimotor information contribution to semantic knowledge, emphasize the contextual relevance of these two sources. Our aim was to test the LASS theory’s prediction that linguistic information will be prioritized during semantic processing and also, to compare the relative contribution of each information type when performing the property verification task. We operationalized these two types of information as cosine distances measured from GloVe embeddings and the Lancaster sensorimotor norms, and modeled single-trial EEG responses as well as reaction times to property words. Our findings show that both measures account for unique EEG and RT variance and suggest distinct, if not entirely independent, contributions of neural mechanisms sensitive to the statistical properties of language and those that come from our interactions with the world and the sensorimotor origins of our conceptual structure. Their changing dynamics on time-sensitive EEG measures also affirm that contextual differences prompt the recruitment of different resources based on the task and linguistic conditions.

## ACKNOWLEDGMENTS

We thank Dr. Jennifer Collins for her contribution to data collection, and Dr. Marta Kutas for her inputs on the manuscript. We are also grateful to the anonymous reviewers for their concrete and insightful feedback that greatly improved the manuscript.

## FUNDING INFORMATION

The authors have no funding to declare.

## AUTHOR CONTRIBUTIONS

H.V.: Conceptualization; Formal analysis; Methodology; Software; Visualization; Writing – original draft; Writing – review & editing. S.T.: Conceptualization; Methodology; Writing – review & editing. D.P.: Conceptualization; Investigation; Writing – review & editing. R.Z.: Conceptualization; Investigation; Writing – review & editing. S.C.: Conceptualization; Methodology; Resources; Supervision; Writing – review & editing.

## DATA AVAILABILITY STATEMENT

The data and code for the findings presented are available in the public repository. Link: https://osf.io/6efp8.

## Note

^1^ In this paper, we refer to concepts via words in all capitals (e.g., COW); we refer to the representation in the mental lexicon via lowercase letters (e.g., cow); and we refer to the printed word form via quotation marks (e.g., “cow”). As our study employed words referring both to concepts and their associated properties, we used all capitals to depict the concept terms and lowercase letters to depict the property words. Consequently, when referring to materials viewed by participants, we mark concept words using capital letters within quotation marks (e.g., “APPLE”), and property words in lowercase letters within quotation marks (e.g., “red”).

## Supplementary Material



## References

[bib2] Auguste, J., Rey, A., & Favre, B. (2017). Evaluation of word embeddings against cognitive processes: Primed reaction times in lexical decision and naming tasks. In S. Bowman, Y. Goldberg, F. Hill, A. Lazaridou, O. Levy, R. Reichart, & A. Søgaard (Eds.), Proceedings of the 2nd Workshop on Evaluating Vector Space Representations for NLP (pp. 21–26). Association for Computational Linguistics. 10.18653/v1/W17-5304

[bib3] Bardolph, M., & Coulson, S. (2014). How vertical hand movements impact brain activity elicited by literally and metaphorically related words: An ERP study of embodied metaphor. Frontiers in Human Neuroscience, 8, 1031. 10.3389/fnhum.2014.01031, 25566041 PMC4274969

[bib4] Barsalou, L. W. (2015). Situated conceptualization: Theory and applications. In Y. Coello & M. H. Fischer (Eds.), Perceptual and emotional embodiment: Foundations of embodied cognition (Vol. 1, pp. 11–37). Routledge/Taylor & Francis Group.

[bib5] Barsalou, L. W., Santos, A., Simmons, W. K., & Wilson, C. D. (2008). Language and simulation in conceptual processing. In M. de Vega, A. Glenberg, & A. Graesser (Eds.), Symbols and embodiment: Debates on meaning and cognition. Oxford University Press. 10.1093/acprof:oso/9780199217274.003.0013

[bib6] Barsalou, L. W., Simmons, W. K., Barbey, A. K., & Wilson, C. D. (2003). Grounding conceptual knowledge in modality-specific systems. Trends in Cognitive Sciences, 7(2), 84–91. 10.1016/s1364-6613(02)00029-3, 12584027

[bib7] Bates, D., Mächler, M., Bolker, B., & Walker, S. (2015). Fitting linear mixed-effects models using lme4. Journal of Statistical Software, 67(1), 1–48. 10.18637/jss.v067.i01

[bib8] Beltrán, D., Muñetón-Ayala, M., & de Vega, M. (2018). Sentential negation modulates inhibition in a stop-signal task. Evidence from behavioral and ERP data. Neuropsychologia, 112, 10–18. 10.1016/j.neuropsychologia.2018.03.004, 29518413

[bib9] Benjamini, Y., & Yekutieli, D. (2001). The control of the false discovery rate in multiple testing under dependency. Annals of Statistics, 29(4), 1165–1188. 10.1214/aos/1013699998

[bib10] Bi, Y. (2021). Dual coding of knowledge in the human brain. Trends in Cognitive Sciences, 25(10), 883–895. 10.1016/j.tics.2021.07.006, 34509366

[bib11] Binder, J. R., Conant, L. L., Humphries, C. J., Fernandino, L., Simons, S. B., Aguilar, M., & Desai, R. H. (2016). Toward a brain-based componential semantic representation. Cognitive Neuropsychology, 33(3–4), 130–174. 10.1080/02643294.2016.1147426, 27310469

[bib12] Binder, J. R., & Desai, R. H. (2011). The neurobiology of semantic memory. Trends in Cognitive Sciences, 15(11), 527–536. 10.1016/j.tics.2011.10.001, 22001867 PMC3350748

[bib13] Brysbaert, M., New, B., & Keuleers, E. (2012). Adding part-of-speech information to the SUBTLEX-US word frequencies. Behavior Research Methods, 44(4), 991–997. 10.3758/s13428-012-0190-4, 22396136

[bib14] Burnham, K. P., & Anderson, D. R. (2004). Multimodel inference: Understanding AIC and BIC in model selection. Sociological Methods & Research, 33(2), 261–304. 10.1177/0049124104268644

[bib15] Carpenter, P. A., & Just, M. A. (1975). Sentence comprehension: A psycholinguistic processing model of verification. Psychological Review, 82(1), 45–73. 10.1037/h0076248

[bib16] Collins, J., Pecher, D., Zeelenberg, R., & Coulson, S. (2011). Modality switching in a property verification task: An ERP study of what happens when candles flicker after high heels click. Frontiers in Psychology, 2, 10. 10.3389/fpsyg.2011.00010, 21713128 PMC3111443

[bib17] Connell, L. (2019). What have labels ever done for us? The linguistic shortcut in conceptual processing. Language, Cognition and Neuroscience, 34(10), 1308–1318. 10.1080/23273798.2018.1471512

[bib18] Connell, L., & Lynott, D. (2011). Modality switching costs emerge in concept creation as well as retrieval. Cognitive Science, 35(4), 763–778. 10.1111/j.1551-6709.2010.01168.x, 21564270

[bib19] Connell, L., & Lynott, D. (2013). Flexible and fast: Linguistic shortcut affects both shallow and deep conceptual processing. Psychonomic Bulletin & Review, 20(3), 542–550. 10.3758/s13423-012-0368-x, 23307559

[bib20] Courellis, H. S., Iversen, J. R., Poizner, H., & Cauwenberghs, G. (2016). EEG channel interpolation using ellipsoid geodesic length. In 2016 IEEE Biomedical Circuits and Systems Conference (BioCAS) (pp. 540–543). IEEE. 10.1109/BioCAS.2016.7833851

[bib21] Davis, C. P., & Yee, E. (2021). Building semantic memory from embodied and distributional language experience. Wiley Interdisciplinary Reviews: Cognitive Science, 12(5), e1555. 10.1002/wcs.1555, 33533205

[bib22] Dufau, S., Grainger, J., Midgley, K. J., & Holcomb, P. J. (2015). A thousand words are worth a picture: Snapshots of printed-word processing in an event-related potential megastudy. Psychological Science, 26(12), 1887–1897. 10.1177/0956797615603934, 26525074 PMC4679424

[bib23] Emmorey, K., Winsler, K., Midgley, K. J., Grainger, J., & Holcomb, P. J. (2020). Neurophysiological correlates of frequency, concreteness, and iconicity in American Sign Language. Neurobiology of Language, 1(2), 249–267. 10.1162/nol_a_00012, 33043298 PMC7544239

[bib24] Ettinger, A., & Linzen, T. (2016). Evaluating vector space models using human semantic priming results. In Proceedings of the 1st Workshop on Evaluating Vector-Space Representations for NLP (pp. 72–77). Association for Computational Linguistics. 10.18653/v1/W16-2513

[bib26] Fernandino, L., Humphries, C. J., Conant, L. L., Seidenberg, M. S., & Binder, J. R. (2016). Heteromodal cortical areas encode sensory-motor features of word meaning. Journal of Neuroscience, 36(38), 9763–9769. 10.1523/JNEUROSCI.4095-15.2016, 27656016 PMC5030346

[bib27] Fernandino, L., Tong, J.-Q., Conant, L. L., Humphries, C. J., & Binder, J. R. (2022). Decoding the information structure underlying the neural representation of concepts. Proceedings of the National Academy of Sciences, 119(6), e2108091119. 10.1073/pnas.2108091119, 35115397 PMC8832989

[bib28] Fischler, I., Bloom, P. A., Childers, D. G., Arroyo, A. A., & Perry, N. W., Jr. (1984). Brain potentials during sentence verification: Late negativity and long-term memory strength. Neuropsychologia, 22(5), 559–568. 10.1016/0028-3932(84)90020-4, 6504296

[bib29] Fox, J., & Weisberg, S. (2019). An R companion to applied regression (3rd ed.). Sage. https://www.john-fox.ca/Companion/

[bib31] Gramfort, A., Luessi, M., Larson, E., Engemann, D. A., Strohmeier, D., Brodbeck, C., Goj, R., Jas, M., Brooks, T., Parkkonen, L., & Hämäläinen, M. (2013). MEG and EEG data analysis with MNE-Python. Frontiers in Neuroscience, 7, 267. 10.3389/fnins.2013.00267, 24431986 PMC3872725

[bib32] Hald, L. A., Hocking, I., Vernon, D., Marshall, J.-A., & Garnham, A. (2013). Exploring modality switching effects in negated sentences: Further evidence for grounded representations. Frontiers in Psychology, 4, 93. 10.3389/fpsyg.2013.00093, 23450002 PMC3584287

[bib33] Harris, Z. S. (1954). Distributional structure. Word, 10(2–3), 146–162. 10.1080/00437956.1954.11659520

[bib34] Hauk, O., Johnsrude, I., & Pulvermüller, F. (2004). Somatotopic representation of action words in human motor and premotor cortex. Neuron, 41(2), 301–307. 10.1016/s0896-6273(03)00838-9, 14741110

[bib35] Hauk, O., & Pulvermüller, F. (2004). Effects of word length and frequency on the human event-related potential. Clinical Neurophysiology, 115(5), 1090–1103. 10.1016/j.clinph.2003.12.020, 15066535

[bib36] Hauk, O., & Tschentscher, N. (2013). The body of evidence: What can neuroscience tell us about embodied semantics? Frontiers in Psychology, 4, 50. 10.3389/fpsyg.2013.00050, 23407791 PMC3570773

[bib38] Hoenig, K., Sim, E.-J., Bochev, V., Herrnberger, B., & Kiefer, M. (2008). Conceptual flexibility in the human brain: Dynamic recruitment of semantic maps from visual, motor, and motion-related areas. Journal of Cognitive Neuroscience, 20(10), 1799–1814. 10.1162/jocn.2008.20123, 18370598

[bib39] Kan, I. P., Barsalou, L. W., Solomon, K. O., Minor, J. K., & Thompson-Schill, S. L. (2003). Role of mental imagery in a property verification task: FMRI evidence for perceptual representations of conceptual knowledge. Cognitive Neuropsychology, 20(3–6), 525–540. 10.1080/02643290244000257, 20957583

[bib40] Kappenman, E. S., & Luck, S. J. (2012). ERP components: The ups and downs of brainwave recordings. In E. S. Kappenman & S. J. Luck (Eds.), The Oxford handbook of event-related potential components (pp. 4–30). Oxford University Press. 10.1093/oxfordhb/9780195374148.013.0014

[bib42] Kemmerer, D. (2019). Concepts in the brain: The view from cross-linguistic diversity. Oxford University Press. 10.1093/oso/9780190682620.001.0001

[bib43] Kiefer, M., Sim, E.-J., Liebich, S., Hauk, O., & Tanaka, J. (2007). Experience-dependent plasticity of conceptual representations in human sensory-motor areas. Journal of Cognitive Neuroscience, 19(3), 525–542. 10.1162/jocn.2007.19.3.525, 17335399

[bib44] Kuhnke, P., Kiefer, M., & Hartwigsen, G. (2020). Task-dependent recruitment of modality-specific and multimodal regions during conceptual processing. Cerebral Cortex, 30(7), 3938–3959. 10.1093/cercor/bhaa010, 32219378 PMC7264643

[bib45] Kutas, M., & Federmeier, K. D. (2011). Thirty years and counting: Finding meaning in the N400 component of the event-related brain potential (ERP). Annual Review of Psychology, 62, 621–647. 10.1146/annurev.psych.093008.131123, 20809790 PMC4052444

[bib46] Kuznetsova, A., Brockhoff, P. B., & Christensen, R. H. B. (2017). lmerTest package: Tests in linear mixed effects models. Journal of Statistical Software, 82(13), 1–26. 10.18637/jss.v082.i13

[bib47] Landauer, T. K., & Dumais, S. T. (1997). A solution to Plato’s problem: The latent semantic analysis theory of acquisition, induction, and representation of knowledge. Psychological Review, 104(2), 211–240. 10.1037/0033-295X.104.2.211

[bib48] Lenci, A. (2018). Distributional models of word meaning. Annual Review of Linguistics, 4, 151–171. 10.1146/annurev-linguistics-030514-125254

[bib50] Louwerse, M. M. (2008). Embodied relations are encoded in language. Psychonomic Bulletin & Review, 15(4), 838–844. 10.3758/PBR.15.4.838, 18792513

[bib51] Louwerse, M. M. (2011). Symbol interdependency in symbolic and embodied cognition. Topics in Cognitive Science, 3(2), 273–302. 10.1111/j.1756-8765.2010.01106.x, 25164297

[bib52] Louwerse, M. M., & Jeuniaux, P. (2010). The linguistic and embodied nature of conceptual processing. Cognition, 114(1), 96–104. 10.1016/j.cognition.2009.09.002, 19818435

[bib53] Luck, S. J. (2014). An introduction to the event-related potential technique (2nd ed.). MIT Press.

[bib54] Lynott, D., Connell, L., Brysbaert, M., Brand, J., & Carney, J. (2020). The Lancaster Sensorimotor Norms: Multidimensional measures of perceptual and action strength for 40,000 English words. Behavior Research Methods, 52(3), 1271–1291. 10.3758/s13428-019-01316-z, 31832879 PMC7280349

[bib55] Mandera, P., Keuleers, E., & Brysbaert, M. (2017). Explaining human performance in psycholinguistic tasks with models of semantic similarity based on prediction and counting: A review and empirical validation. Journal of Memory and Language, 92, 57–78. 10.1016/j.jml.2016.04.001

[bib56] Meteyard, L., Cuadrado, S. R., Bahrami, B., & Vigliocco, G. (2012). Coming of age: A review of embodiment and the neuroscience of semantics. Cortex, 48(7), 788–804. 10.1016/j.cortex.2010.11.002, 21163473

[bib57] Michaelov, J. A., Bardolph, M. D., Van Petten, C. K., Bergen, B. K., & Coulson, S. (2024). Strong prediction: Language model surprisal explains multiple N400 effects. Neurobiology of Language, 5(1), 107–135. 10.1162/nol_a_00105, 38645623 PMC11025652

[bib58] Mikolov, T., Chen, K., Corrado, G., & Dean, J. (2013). Efficient estimation of word representations in vector space. arXiv. 10.48550/arXiv.1301.3781

[bib60] Pecher, D., Zeelenberg, R., & Barsalou, L. W. (2003). Verifying different-modality properties for concepts produces switching costs. Psychological Science, 14(2), 119–124. 10.1111/1467-9280.t01-1-01429, 12661672

[bib61] Pennington, J., Socher, R., & Manning, C. (2014). GloVe: Global vectors for word representation. In A. Moschitti, B. Pang, & W. Daelemans (Eds.), Proceedings of the 2014 Conference on Empirical Methods in Natural Language Processing (EMNLP) (pp. 1532–1543). Association for Computational Linguistics. 10.3115/v1/D14-1162

[bib1] Penolazzi, B., Hauk, O., & Pulvermüller, F. (2007). Early semantic context integration and lexical access as revealed by event-related brain potentials. Biological Psychology, 74(3), 374–388. 10.1016/j.biopsycho.2006.09.008, 17150298

[bib62] Pulvermüller, F. (2005). Brain mechanisms linking language and action. Nature Reviews Neuroscience, 6(7), 576–582. 10.1038/nrn1706, 15959465

[bib63] Pulvermüller, F., Härle, M., & Hummel, F. (2001). Walking or talking? Behavioral and neurophysiological correlates of action verb processing. Brain and Language, 78(2), 143–168. 10.1006/brln.2000.2390, 11500067

[bib64] Raji, S., & de Melo, G. (2020). What sparks joy: The AffectVec emotion database. In Y. Huang, I. King, T.-Y. Liu, & M. van Steen (Eds.), Proceedings of The Web Conference 2020 (pp. 2991–2997). Association for Computing Machinery. 10.1145/3366423.3380068

[bib65] Reilly, J., Finley, A. M., Litovsky, C. P., & Kenett, Y. N. (2023). Bigram semantic distance as an index of continuous semantic flow in natural language: Theory, tools, and applications. Journal of Experimental Psychology: General, 152(9), 2578–2590. 10.1037/xge0001389, 37079833 PMC10790181

[bib66] Richie, R., & Bhatia, S. (2021). Similarity judgment within and across categories: A comprehensive model comparison. Cognitive Science, 45(8), e13030. 10.1111/cogs.13030, 34379325

[bib67] Simmons, W. K., Hamann, S. B., Harenski, C. L., Hu, X. P., & Barsalou, L. W. (2008). fMRI evidence for word association and situated simulation in conceptual processing. Journal of Physiology-Paris, 102(1–3), 106–119. 10.1016/j.jphysparis.2008.03.014, 18468869

[bib68] Smith, N. J., & Kutas, M. (2015). Regression-based estimation of ERP waveforms: I. The rERP framework. Psychophysiology, 52(2), 157–168. 10.1111/psyp.12317, 25141770 PMC5308234

[bib69] Solomon, K. O., & Barsalou, L. W. (2004). Perceptual simulation in property verification. Memory & Cognition, 32(2), 244–259. 10.3758/BF03196856, 15190717

[bib71] Tremblay, A., & Newman, A. J. (2015). Modeling nonlinear relationships in ERP data using mixed-effects regression with R examples. Psychophysiology, 52(1), 124–139. 10.1111/psyp.12299, 25132114

[bib72] Trott, S., & Bergen, B. (2021). RAW-C: Relatedness of ambiguous words in context (a new lexical resource for English). In C. Zong, F. Xia, W. Li, & R. Navigli (Eds.), Proceedings of the 59th Annual Meeting of the Association for Computational Linguistics and the 11th International Joint Conference on Natural Language Processing (Volume 1: Long Papers) (pp. 7077–7087). Association for Computational Linguistics. 10.18653/v1/2021.acl-long.550

[bib73] Trott, S., & Bergen, B. (2022). Contextualized Sensorimotor Norms: Multi-dimensional measures of sensorimotor strength for ambiguous English words, in context. arXiv. 10.48550/arXiv.2203.05648 PMC13469423 42587187

[bib75] Wang, X., Men, W., Gao, J., Caramazza, A., & Bi, Y. (2020). Two forms of knowledge representations in the human brain. Neuron, 107(2), 383–393. 10.1016/j.neuron.2020.04.010, 32386524

[bib76] Wason, P. C. (1961). Response to affirmative and negative binary statements. British Journal of Psychology, 52(2), 133–142. 10.1111/j.2044-8295.1961.tb00775.x, 13783300

[bib77] Wingfield, C., & Connell, L. (2023). Sensorimotor distance: A grounded measure of semantic similarity for 800 million concept pairs. Behavior Research Methods, 55(7), 3416–3432. 10.3758/s13428-022-01965-7, 36131199 PMC10615916

[bib79] Winkielman, P., Coulson, S., & Niedenthal, P. (2018). Dynamic grounding of emotion concepts. Philosophical transactions of the Royal Society of London, Series B: Biological Sciences, 373(1752), 20170127. 10.1098/rstb.2017.0127, 29914995 PMC6015836

[bib80] Winkielman, P., Davis, J. D., & Coulson, S. (2023). Moving thoughts: Emotion concepts from the perspective of context dependent embodied simulation. Language, Cognition and Neuroscience, 38(10), 1531–1553. 10.1080/23273798.2023.2236731

[bib81] Winsler, K., Holcomb, P. J., & Emmorey, K. (2023). Electrophysiological patterns of visual word recognition in deaf and hearing readers: An ERP mega-study. Language, Cognition and Neuroscience, 38(5), 636–650. 10.1080/23273798.2022.2135746, 37304206 PMC10249718

[bib82] Winsler, K., Midgley, K. J., Grainger, J., & Holcomb, P. J. (2018). An electrophysiological megastudy of spoken word recognition. Language, Cognition and Neuroscience, 33(8), 1063–1082. 10.1080/23273798.2018.1455985, 33912620 PMC8078007

[bib83] Wixted, J. T. (2007). Dual-process theory and signal-detection theory of recognition memory. Psychological Review, 114(1), 152–176. 10.1037/0033-295X.114.1.152, 17227185

[bib84] Yan, S., & Jaeger, T. F. (2020). (Early) context effects on event-related potentials over natural inputs. Language, Cognition and Neuroscience, 35(5), 658–679. 10.1080/23273798.2019.1597979, 32617349 PMC7331969

[bib85] Yang, C. L., & Perfetti, C. A. (2006). Contextual constraints on the comprehension of relative clause sentences in Chinese: ERPs evidence. Language and Linguistics, 7(3), 697–730.

[bib86] Yonelinas, A. P. (1994). Receiver-operating characteristics in recognition memory: Evidence for a dual-process model. Journal of Experimental Psychology: Learning, Memory, and Cognition, 20(6), 1341–1354. 10.1037/0278-7393.20.6.1341, 7983467

