# Supplementary material for: Words and Worlds Both: Dynamic Effects of Distributional and Sensorimotor Information in Semantic Processing
Source: Open Mind (Camb). 2025 Dec 18;9:2149–74. doi: 10.1162/OPMI.a.316 (PMC12768550; doi:10.1162/OPMI.a.316)
Supplement: Supplementary file 1 [file opmi-09-2149-s001.pdf]

Supplementary Materials

1. Breaking down the EEG regression model structure:

Let's take the Baseline model for example. Its structure is:

Voltage = Intercept + X + Y + Z + word_frequency
+ word_frequency:X + word_frequency:Y + word_frequency:Z
+ (1 | subject) + (1 | word) **— (1)**

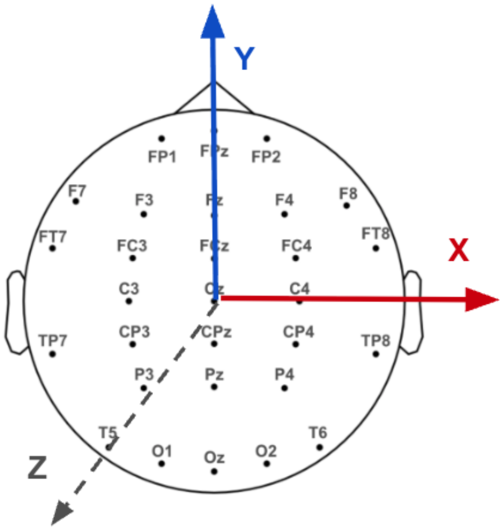

Figure 1: Visualization of X, Y, Z scalp dimensions and how they vary across the electrodes

Here, X, Y, and Z are continuous predictors representing the scalp dimensions across which the electrodes vary (Figure 1). Such that each electrode has its unique combination of the X, Y and Z coordinates.

For example, FP1's location is [-0.027, 0.082, -0.003], where -0.027 marks its location on the horizontal (left to right) X axis, and so on. And Cz, as obvious from the figure has [0, 0, 0]. Thus, the predictor X is a continuous variable maintaining the X axis location values for all the 29 electrodes; similarly for the Y and Z axes predictors. In the above equation, the parameter "word_frequency:X" captures how the effect of word frequency changes from left side of the scalp to right. Conceptualizing EEG channels as either random or as different levels of a fixed effect poses challenges due to correlations between adjacent channels, such that data from two adjacent channels in either of these approaches get treated as independent observations. Therefore, we aimed to enhance interpretability by adopting the modeling approach using the X, Y and Z scalp topography dimensions (borrowing the method from Winsler et al., 2018).

2. Estimates for all RTs models

The two tables below summarize the mixed effects models of RTs reported in the main text. Each table lists fixed effect estimates and corresponding p-values for each of the four RT models of TRUE trials in (a) and of FALSE trials in (b).

RTs model estimates:	(a) TRUE trials							
	Base model		Distributional model D		Sensorimotor model S		Combined model DS	
Predictors	estimates	p-values	estimates	p-values	estimates	p-values	estimates	p-values
Intercept	971.48	<0.001	982.33	<0.001	975.7	<0.001	983.37	<0.001
Word Frequency	-35.54	<0.001	-35.0	<0.001	-36.14	<0.001	-35.26	<0.001
Distributional	-	-	22.74	0.003	-	-	21.58	0.006
Sensorimotor	-	-	-	-	13.91	0.172	5.24	0.619
	(b) FALSE trials							
	Base model		Distributional model D		Sensorimotor model S		Combined model DS	
Predictors	estimates	p-values	estimates	p-values	estimates	p-values	estimates	p-values
Intercept	953.86	<0.001	965.42	<0.001	959.67	<0.001	969.24	<0.001
Word Frequency	-2.60	0.781	-6.88	0.455	-4.1	0.637	-7.67	0.375
Distributional			-27.71	0.002			-23.73	0.006
Sensorimotor					-29.3	<0.001	-27.25	<0.001

3. Estimation of coefficients in Figure 7 of the manuscript:

The formula $[\beta + \beta x \cdot Xi + \beta y \cdot Yi + \beta z \cdot Zi]$ is employed to estimate the effect of each predictor at individual channel level. For example, word_frequency at channel FP1. Let's take the Baseline model's equation (1) described in the first section in this document.

Based on the equation, we get four coefficients for word_frequency's effect from our model structure: the predictor's main effect, and how the predictor's effect varies across

the X scalp dimension, how it varies across the Y scalp dimension, and across the Z scalp dimension. These four estimates, while stable, are insufficient on their own to represent the topographic effects familiar to classical ERP literature. The goal for this formula ($\beta + \beta x \cdot Xi + \beta y \cdot Yi + \beta z \cdot Zi$) is to utilize the four coefficients (i.e., β, βx, βy and βz) we get for each predictor and estimate a channel level effect by scaling them for each channel's coordinates. So for effect of word_frequency in the Baseline model at channel FP1 [-0.027, 0.082, -0.003], we multiply the coefficient of "word_frequency:X" by -0.027, viz., the X coordinate value of FP1; we then multiply the coefficient of word_frequency:Y by 0.082 (the Y coordinate value of FP1), and the coefficient of word_frequency:Z by -0.003 (the Z coordinate), and sum these three products with the coefficient of the main effect of "word_frequency" to get the estimated effect of word frequency at FP1.

4. Topography of the effects from the best models.

Figure 7 in the main paper presents topographies of estimated beta values from the combined model— the Distributional and Sensorimotor (DS) model EEG data, with a goal to present the estimated effects when each predictor is competing with all other predictors. However, to facilitate comparison with Figure 7 in the paper, we report beta estimates from the best-fitting models in each analysis window (Figure 2).

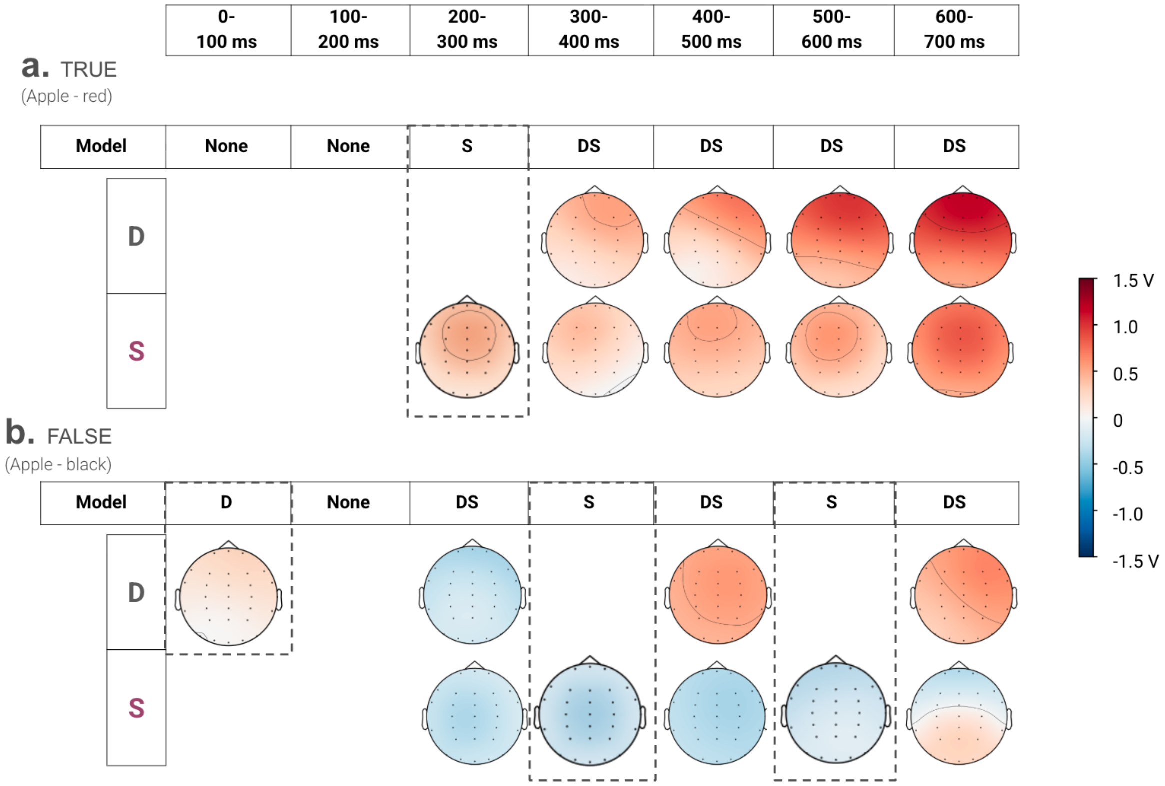

Figure 2: This figure shows topographic distribution of beta values from the D, S and DS models based on which amongst them provided a robustly better fit (ΔAIC ≥ 10) than the others. 'None' suggests the Base model, with only word frequency, provides the best fit to the data. Because word frequency was a control predictor and was included in all models, it is not included in this figure. The estimates at each channel are derived from the process explained in section 2 above. We note that the pattern of distributional and sensorimotor distance effects here resembles those depicted in Figure 7 in the main text suggesting the estimates of both effects are stable.
